# External quality assurance for HIV point-of-care testing in Africa: A collaborative country-partner approach to strengthen diagnostic services

**DOI:** 10.4102/ajlm.v5i2.556

**Published:** 2016-10-17

**Authors:** Debrah I. Boeras, Rosanna W. Peeling

**Affiliations:** 1 London School of Hygiene and Tropical Medicine, London, United Kingdom

## Abstract

It is important to consider the role of diagnostics and the critical need for quality diagnostics services in resource-limited settings. Accurate diagnostic tests play a key role in patient management and the prevention and control of most infectious diseases. As countries plan for implementation of HIV early infant diagnosis and viral load point-of-care testing, the London School of Hygiene & Tropical Medicine has worked with countries and partners with an interest in external quality assurance to support quality point-of-care testing on the continent. Through a series of collaborative consultations and workshops, the London School of Hygiene & Tropical Medicine has gathered lessons learned, tools, and resources and developed quality assurance models that will support point-of-care testing. The London School of Hygiene & Tropical Medicine is committed to the continued advancement of laboratory diagnostics in Africa and quality laboratory services and point-of-care testing.

## Diagnostic landscape in the developing world

Accurate diagnostic tests play a key role in patient management and the prevention and control of most infectious diseases (Table 1). Early diagnosis and treatment not only reduces the risk of developing long-term complications in the patient, but for diseases such as tuberculosis, sexually-transmitted infections and HIV, prompt treatment also reduces further transmission to other members of the community.

**TABLE 1:** Role of diagnostic tests in patient management and disease control.

Aspects of patient management and disease control	Role of diagnostic tests
Case management	Screening for asymptomatic infections
	Diagnosis and/or staging of infections when clinical presentation is non-specific
Disease control	Surveillance
	Outbreak investigations
	Monitoring the effectiveness of interventions
Elimination	Certifying elimination
Treatment efficacy	Detecting and monitoring drug resistance

In many developing countries, laboratory services are often limited to major urban centres. In settings where access to diagnostic laboratory services is limited, the World Health Organization (WHO) recommends the use of a syndromic approach to clinical management, where patients presenting with a particular syndrome are treated for all the major causes of the syndrome.

Although these algorithms are inexpensive and easy to use, especially in primary healthcare settings, a major disadvantage is over-treatment and the potential for development of antibiotic resistance. Syndromic management also does not allow contact tracing to prevent onward transmission, public health surveillance and interventions.

Even when laboratory services are available, there are often problems with the quality of the services due to lack of resources, supply logistics and trained personnel. These problems lead to physicians not trusting laboratory results, which in turn leads to further neglect of laboratory services.

With the more recent Joint United Nations Programme on HIV/AIDS 90-90-90 targets to identify 90% of those HIV infected, provide treatment to 90% of those HIV infected, and ensure viral suppression for 90% of those on treatment, diagnostics near the point of care will play a pivotal role for expanding HIV diagnosis and monitoring.^1^

### Lack of international and national regulatory standards for approval of diagnostics

National regulatory processes for drugs provide safeguards for the safety and efficacy of drugs used in a country. Most countries have a process for reviewing the evidence from drug trials to support the introduction of new drugs, and this has done much to improve the quality of therapeutics used in developing countries. Unfortunately, apart from tests used for blood banking, regulatory standards are often lacking for diagnostic tests, especially those targeting diseases that are uncommon in industrialised countries. As a result, diagnostic tests are often sold in most of the developing world, without any formal evaluation of their performance and effectiveness.

## Ten years of building capacity to assure quality

In a review article by Petti et al. in 2006, the authors described laboratory medicine as a barrier to effective healthcare, with misdiagnosis occurring often.^2^ Clinicians must rely on alternative diagnostics in their clinical practice due to the lack of available and affordable diagnostics. This article elicited numerous responses, acknowledging diagnostic insufficiencies in Africa, and even referring to laboratory systems and diagnostics as the Achilles Heel of global health.^3,4^ As of 10 years ago, we understood the need for sustainable laboratory infrastructures to support the diagnosis of infectious disease.

**FIGURE 1:**
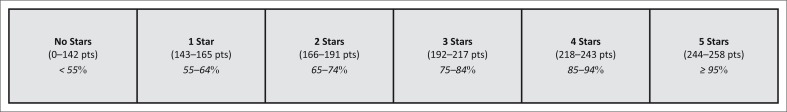
Stepwise Laboratory Quality Improvement Process Towards Accreditation (SLIPTA) inspection checklist score corresponds to the number of stars awarded to a laboratory.

Since 2008, there have been several landmark events to advance laboratory diagnostics in Africa:

- 2008: January – the Maputo Declaration for strengthening laboratory health systems;^5^ April – the Lyon Statement on the need for developing countries to establish practical quality management systems;^6^ and September – the Yaoundé resolution issued by the WHO Regional Office for Africa (WHO AFRO) to strengthen public health laboratories in the WHO African region;^7^
- July 2009: Several African countries, donors, WHO and implementing partners met in Kigali, Rwanda, to launch the Stepwise Laboratory Quality Improvement Process Towards Accreditation (SLIPTA), the WHO AFRO accreditation scheme to assist laboratories in obtaining internationally-recognised accreditation standards^8^ (ISO – International Organization for Standardization; CAP – College of American Pathologists; SANAS – South African National Accreditation System; SADCAS – Southern African Development Community Accreditation Service);
- 2010: The Kampala Statement called for the creation of the African Society for Laboratory Medicine;^9^
- 2011: Launch of the African Society for Laboratory Medicine as a pan-African professional body working to advocate for the critical role and needs of laboratory medicine and networks throughout Africa. The African Society for Laboratory Medicine addresses these challenges by working collaboratively with governments, local and international organisations, implementing partners, and the private sector to achieve the following goals by 2020:
  ■ Strengthening laboratory workforce by training and certifying laboratory professionals and clinicians through standardised frameworks;
  ■ Transforming laboratory testing quality by enrolling laboratories in quality improvement programmes to achieve accreditation by international standards;
  ■ Developing strong, harmonised regulatory systems for diagnostic products as defined by the Global Harmonization Taskforce; and
  ■ Building a network of national public health reference laboratories to improve early disease detection and collaborative research.
- 2015: WHO/US Centers for Disease Control and Prevention point-of-care (POC) handbook;^10^
- 2016: 27 of the 47 (57%) WHO AFRO member states had appointed a SLIPTA focal point and 14 Ministers of Health had endorsed SLIPTA as the desired programme for continuous quality improvement. 159 laboratories were audited between May 2013 and March 2015, with a 69% mean score. Of those audited, 70% achieved 55% compliance or higher (2 or more stars) and 1% scored at least 95% (5 stars). The lowest scoring sections of the WHO AFRO SLIPTA checklist were sections 6 (Internal Audit) and 10 (Corrective Action), which both had mean scores below 50%.^8^ Figure 1 shows the scoring system (stars) as outlined by the SLIPTA inspection checklist.

This progress is very encouraging, but there are new challenges with the introduction of POC testing:

- POC testing is performed and results interpreted by non-lab personnel with no expertise or experience in doing any form of testing;
- POC devices and cartridges are transported all over the country without temperature control;
- POC devices and cartridges stored at remote sites often have no temperature control. The devices may be dusty or exposed to extreme humidity. All of these affect the test validity;
- Many POC tests lack ‘on-board’ controls, meaning that there is no quality control for the testing process;
- Central or national laboratories often do not have sufficient capacity to supervise all POC testing or institute external quality assessment (EQA) programmes for every POC site.

### A collaborative approach to assure quality

In consultation with the Ministry of Health and representatives from 10 countries with an interest in external quality assurance, the London School of Hygiene and Tropical Medicine, through funding from UNITAID, worked with partners and quality assurance experts to develop strategies and initiatives to support countries in scaling up quality POC testing. Countries shared lessons learned and possible planning for quality POC testing.

### Quality assurance models for point-of-care testing

**FIGURE 2:**
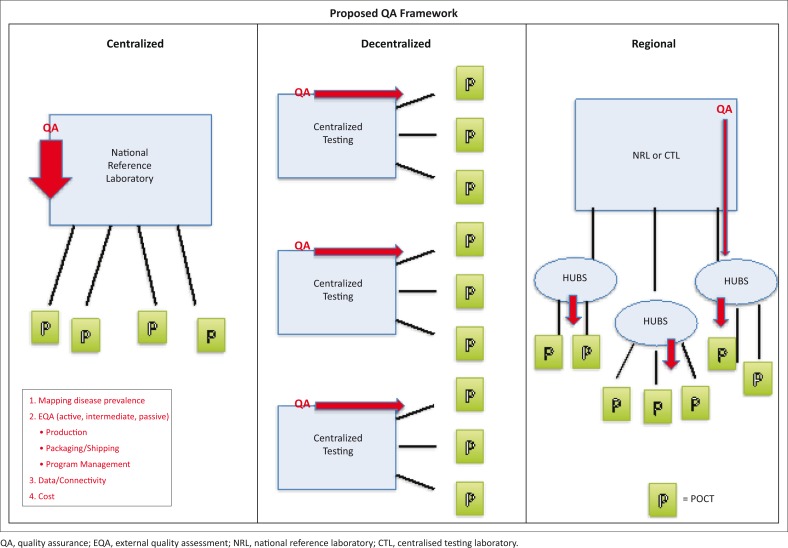
Quality assurance models for point-of-care testing integrated within existing laboratory networks.

While POC has the potential to provide faster results for quicker clinical decisions, a major concern with decentralising diagnostics and laboratory services is the lack of oversight and ensuring quality standards. From the consultations, several models were captured for organising POC testing in Africa (Figure 2).

### Quality assurance/external quality assurance consultations for point-of-care testing

#### Initial country consultations with UNICEF

These consultations found a large need for quality assurance planning and policies, especially around POC testing.

#### Regional country consultations (East and West Africa)

This set the stage for our EQA work, as we found that most countries were enrolled in some sort of EQA programme and were also supporting quality assurance activities. Countries shared lessons and tools and identified major gaps in their quality assurance programmes.

#### US Centers for Disease Control and Prevention, Atlanta, external quality assessment workshops

These brought together EQA providers as a think tank for global proficiency testing schemes, EQA programmes, and capacity building.

#### External quality assessment country pilot

This piloted full coverage of EQA for CD4 POC in one country, which helped the country map the POC landscape and identify gaps and challenges (e.g. broken instruments, supply chain management issues).

#### External quality assessment/Connectivity pilot

This programme explored how to monitor quality assurance of POC testing. It identified numerous gaps as well as promising opportunities with connectivity and automated EQA reporting.

#### Costing workshops

Countries shared costing information and were trained by modellers and economists to perform costing exercises. This information has formed the basis for costing quality assurance as a percentage of the total cost of a POC test. This information can be used for advocacy and budget planning, as well as business plan development.

#### Technical external quality assessment workshops

A series of technical workshops to provide global coverage for HIV CD4, EID, and viral load external quality assurance, including proficiency testing schemes, and to transition EQA programmes to the continent through technology transfer trainings and capacity-building discussions. These workshops will also support regional EQA centres and south-to-south collaborations.

### Conclusions and way forward

Diagnostics have become central to patient management and disease surveillance/control in the developing world. As laboratory testing is pushed to more remote areas, ensuring accurate diagnostic results as well as sustainable laboratory services becomes a greater challenge to systems that are already struggling.

The London School of Hygiene & Tropical Medicine is working to continue the advancement of laboratory diagnostics in Africa, working with African countries and partners with an interest in EQA to support quality POC testing on the continent. Through a series of collaborative consultations and workshops, the London School of Hygiene and Tropical Medicine has gathered lessons learned, tools and resources, and developed quality assurance models that will support POC testing.

It is important to consider the role of diagnostics and the critical need for quality diagnostics services in the more remote resource-limited settings. Clinicians and patients will increasingly depend on POC testing for improved health outcomes. At the same time, these new diagnostics should be seen as opportunities to improve the health of the community with surveillance and disease control programmes.

## References

[1] UNAIDS 90–90–90 – An ambitious treatment target to help end the AIDS epidemic [document on the Internet]. c2014 [cited 2016 Sep 24]. http://www.unaids.org/en/resources/documents/2014/90-90-90

[2] Petti CA, Polage CR, Quinn TC, et al Laboratory medicine in Africa: a barrier to effective health care. Clin Infect Dis. 2006;42(3):377–382. Epub 2005 Dec 20. 16392084 10.1086/499363

[3] Okeke IN Diagnostic insufficiency in Africa. Clin Infect Dis. 2006;42(10):1501–1503. http://dx.doi.org/10.1086/503308 16619170 10.1086/503308

[4] Berkelman R, Cassell G, Specter S, et al The “Achilles heel” of global efforts to combat infectious diseases. Clin Infect Dis. 2006;42(10):1503–1504. http://dx.doi.org/10.1086/504494 10.1086/504494 16619171

[5] World Health Organization’s Regional Office for Africa The Maputo declaration on strengthening of laboratory systems [document on the Internet]. c2008 [cited 2013 Jan 02]. Available from: http://www.who.int/diagnostics_laboratory/Maputo-Declaration_2008.pdf

[6] World Health Organization Joint WHO–CDC conference on health laboratory quality systems. WHO/HSE/IHR/LYO/2008.3 [document on the Internet]. c2008 [cited 2013 Jan 03]. Available from: http://www.who.int/ihr/lyon/report20080409.pdf

[7] Gershy-Damet GM, Rotz P, Cross D, et al The World Health Organization African region laboratory accreditation process: Improving the quality of laboratory systems in the African region. Am J Clin Pathol. 2010;134(3):393–400. http://dx.doi.org/10.1309/AJCPTUUC2V1WJQBM 20716795 10.1309/AJCPTUUC2V1WJQBM

[8] Yao K, Luman ET, SLMTA Collaborating Authors Evidence from 617 laboratories in 47 countries for SLMTA-driven improvement in quality management systems. Afr J Lab Med. 2014;3(2), Art. #262, 11 pages. http://dx.doi.org/10.4102/ajlm.v3i2.262 10.4102/ajlm.v3i2.262 PMC4706175 26753132

[9] African Field Epidemiology Network (AFENET) Kampala statement for the creation of the African society for laboratory medicine. Kampala, Uganda: AFENET; 2010.

[10] World Health Oranization (WHO)/US Centers for Disease Control and Prevention Improving the quality of HIV-related point-of-care testing: ensuring the reliability and accuracy of test results. Geneva, Switzerland: WHO; 2015.

